# Construction and Analysis of Immune Infiltration-Related ceRNA Network for Kidney Stones

**DOI:** 10.3389/fgene.2021.774155

**Published:** 2021-12-06

**Authors:** Yuqi Xia, Xiangjun Zhou, Zehua Ye, Weimin Yu, Jinzhuo Ning, Yuan Ruan, Run Yuan, Fangyou Lin, Peng Ye, Di Zheng, Ting Rao, Fan Cheng

**Affiliations:** Department of Urology, Renmin Hospital of Wuhan University, Wuhan, China

**Keywords:** kidney stones, ceRNA, immune cell infiltration, calcium oxalate, glyoxylate

## Abstract

**Purpose:** Kidney stones is a common medical issue that mediates kidney injury and even kidney function loss. However, the exact pathogenesis still remains unclear. This study aimed to explore the potential competing endogenous RNA (ceRNA)-related pathogenesis of kidney stones and identify the corresponding immune infiltration signature.

**Methods:** One mRNA and one long non-coding RNA (lncRNA) microarray dataset was obtained from the GEO database. Subsequently, we compared differentially expressed mRNAs (DE-mRNAs) and lncRNAs between Randall’s plaques in patients with calcium oxalate (CaOx) stones and controls with normal papillary tissues. lncRNA-targeted miRNAs and miRNA–mRNA pairs were predicted using the online databases. lncRNA-related DE-mRNAs were identified using the Venn method, and GO and KEGG enrichment analyses were subsequently performed. The immune-related lncRNA–miRNA–mRNA ceRNA network was developed. The CIBERSORT algorithm was used to estimate the rate of immune cell infiltration in Randall’s plaques. The ceRNA network and immune infiltration were validated in the glyoxylate-induced hyperoxaluric mouse model and oxalate-treated HK-2 cells.

**Results:** We identified 2,340 DE-mRNAs and 929 DE-lncRNAs between Randall’s plaques in patients with CaOx stones and controls with normal papillary tissues. lncRNA-related DE-mRNAs were significantly enriched in extracellular matrix organization and collagen-containing extracellular matrix, which were associated with kidney interstitial fibrosis. The immune-related ceRNA network included 10 lncRNAs, 23 miRNAs, and 20 mRNAs. Moreover, we found that M2 macrophages and resting mast cells were differentially expressed between Randall’s plaques and normal tissues. Throughout kidney stone development, kidney tubular injury, crystal deposition, collagen fiber deposition, TGF-β expression, infiltration of M1 macrophages, and activation of mast cells were more frequent in glyoxylate-induced hyperoxaluric mice compared with control mice. Nevertheless, M2 macrophage infiltration increased in early stages (day 6) and decreased as kidney stones progressed (day 12). Furthermore, treatment with 0.25 and 0.5 mM of oxalate for 48 h significantly upregulated NEAT1, PVT1, CCL7, and ROBO2 expression levels and downregulated hsa-miR-23b-3p, hsa-miR-429, and hsa-miR-139-5p expression levels in the HK-2 cell line in a dose-dependent manner.

**Conclusion:** We found that significant expressions of ceRNAs (NEAT1, PVT1, hsa-miR-23b-3p, hsa-miR-429, hsa-miR-139-5p, CCL7, and ROBO2) and infiltrating immune cells (macrophages and mast cells) may be involved in kidney stone pathogenesis. These findings provide novel potential therapeutic targets for kidney stones.

## Introduction

Kidney stones are common and have high incidence and recurrence rates. Kidney stone prevalence in China is 6.4% and increases annually worldwide (Zeng et al., 2017; Kittanamongkolchai et al., 2018), inducing a heavy burden on the healthcare system. Calcium oxalate (CaOx) kidney stones, the most common type of kidney stone, can induce urinary tract obstruction, renal tubular injury, interstitium inflammation and fibrosis, and even chronic renal disease (Rule et al., 2011). However, the process of kidney stone formation is complex, and the exact mechanism remains unclear. Currently, Randall’s plaque (RP), the calcium phosphate crystal deposition at the tip of the renal papillae, is considered to be the origin of kidney stones (Daudon et al., 2015). Crystals in supersaturated urine nucleates deposit in the renal papillae and grow gradually, eventually forming kidney stones (Khan and Canales, 2015). Evidence from endoscopic images demonstrated that stones attach to RP, which appeared in approximately half of patients with kidney stones (Pless et al., 2019). Moreover, renal papillae biopsies have shown that RP formation was associated with high urinary calcium levels, acidic urine, and metabolic diseases (Marien and Miller, 2016). Thus, studying RP to explore the potential pathogenesis of kidney stones and effective therapeutic targets is essential.

Non-coding RNAs (ncRNAs) include long non-coding RNAs (lncRNAs), microRNAs (miRNAs), and circular RNAs, which regulate gene expression at transcriptional and post-transcriptional levels without coding proteins (Beermann et al., 2016). Accumulating evidence has shown that the regulation of mRNAs and ncRNAs is essential for kidney stone-induced renal injury, including apoptosis, oxidative stress, inflammation, and interstitial fibrosis (Liu et al., 2019; Li et al., 2020; Zhu et al., 2020). In recent years, a competing endogenous RNAs (ceRNAs) network hypothesis has been proposed. This hypothesis states that RNAs communicate with each other using miRNA response elements (MREs). LncRNAs regulate the function of mRNAs by competitively binding to the corresponding miRNAs through MREs (Salmena et al., 2011). Given their complexity, the dysregulation of lncRNA–miRNA–mRNA networks is closely related to the pathogenesis of acute and chronic kidney injuries, including ischemia-reperfusion injury and unilateral ureteral obstruction (Cheng et al., 2019; Ren et al., 2019). Nevertheless, few studies have concentrated on the ceRNA regulatory network in patients with kidney stones.

Conventionally, the immune system plays a crucial role in the formation and pathogenesis of kidney stones. Throughout kidney stone development, CaOx crystals promote the secretion of inflammatory cytokines and chemokines, possibly recruiting various immune cells to renal interstitium, including neutrophils, macrophages, and T cells (Zhu et al., 2019; Taguchi et al., 2021). The dysfunction of the immune microenvironment in the kidney could not only initiate adverse factors, but also further exacerbate kidney stone formation (Khan et al., 2021). Previous studies have revealed that M2 macrophages can phagocytize and degrade crystals to suppress stone formation and prevent CaOx inflammatory damage (Taguchi et al., 2021). However, the polarization of M1 macrophages induces cell damage and increases stone burden (Taguchi et al., 2021). In this context, another study has shown that aberrant γδT cells were activated and accumulated in CaOx kidney stones in a mouse model (Zhu et al., 2019). Despite the importance of maintaining immune microenvironmental homeostasis, in patients with kidney stones, the landscape of immune cell infiltration has not been fully clarified.

In this study, we compared differentially expressed (DE) mRNAs and lncRNAs between RPs in patients with CaOx stones and controls with normal papillary tissues based on the Gene Expression Omnibus (GEO) database and constructed an immune-related ceRNA network. Subsequently, to the best of our knowledge, we were the first to estimate the rate of immune cell infiltration in RPs. Moreover, we validated the ceRNA network and immune infiltration *in vivo* and *in vitro*. This study aimed to explore the potential ceRNA-related pathogenesis of kidney stones and identify its corresponding immune infiltration signature.

## Materials and Methods

### Data Acquisition and Differential Expression Analysis

The mRNA microarray dataset GSE73680 (Taguchi et al., 2017) and lncRNA microarray dataset GSE117518 (Zhu et al., 2021) were obtained from the GEO database (https://www.ncbi.nlm.nih.gov/geo/). The GSE73680 dataset included 24 RPs from patients with CaOx stones and six controls with normal papillary tissues. The GSE117518 dataset included three RPs from patients with CaOx stones and three controls with normal papillary tissues. The details of both datasets are presented in Table 1. Probe names were transformed into gene symbols according to platform annotation information. Moreover, immune-related genes were obtained from the Immunology Database and Analysis Portal (IMMPORT) database (http://www.immport.org/) (Bhattacharya et al., 2014).

**TABLE 1:** Details of lncRNA and mRNA datasets of patients with calcium oxalate kidney stones.

Type	GEO accession	Platform	Sample organism	Samples (kidney tissues), *n*		Contributors. (Year)
				Randall’s plaque	Normal papillary	
mRNA	GSE73680	GPL17077	*Homo sapiens*	24	6	Taguchi et al. (2015)
lncRNA	GSE117518	GPL21827	*Homo sapiens*	3	3	Cui et al. (2016)

Subsequently, DE-mRNAs and lncRNAs were analyzed and compared between RPs and normal–papillary tissue controls using the “limma” package (Ritchie et al., 2015) in the R software (http://www.r-project.org). mRNAs that met the criteria of |log_2_FC|>1 and *p* < 0.01 were considered as DE-mRNAs, and lncRNAs that met the criteria of |log_2_FC|>0.58 and *p* < 0.01 were considered as DE-lncRNA. The “ggplot2” package was used to draw heatmaps and volcano plots for data visualization.

### Prediction of lncRNA–miRNA and miRNA–mRNA Interactions

Potential DE-lncRNA-targeted miRNAs were predicted using the miRcode database (http://mircode.org/) (Jeggari et al., 2012). Subsequently, miRNA–mRNA pairs were analyzed using TargetScan (http://www.targetscan.org/vert_72/) (Agarwal et al., 2015), miRTarBase (https://mirtarbase.cuhk.edu.cn/php/index.php) (Huang et al., 2020), and miRDB (http://mirdb.org/) (Chen and Wang, 2020) databases. mRNAs that were found in at least two databases were considered as candidate targets of miRNAs.

### Venn Method

The Venn method was used to analyze overlapping genes. Intersections between DE-mRNA and DE-lncRNA-targeted mRNAs, as well as lncRNA-related DE-mRNAs and immune-related genes were identified using the Venny version 2.1 online tool (https://bioinfogp.cnb.csic.es/tools/venny/index.html).

### Functional Enrichment and Protein–Protein Interaction Analysis

To explore the functions of lncRNA-related DE-mRNAs, gene ontology (GO) and Kyoto Encyclopedia of Genes and Genomes (KEGG) enrichment analyses were conducted using the “org.Hs.eg.db” and “ClusterProfiler” packages (Yu et al., 2012) in the R software. An adjusted *p* < 0.05 was considered statistically significant. Subsequently, the STRING database (https://string-db.org/) (Szklarczyk et al., 2019) was used to determine the relationship between the DE-mRNAs, and Cytoscape software (https://cytoscape.org) was used to develop the PPI network.

### Construction of the Immune-Related ceRNA Network

After identifying immune-related and lncRNA-related DE-mRNAs, the interaction between lncRNAs, miRNAs, and mRNAs was confirmed as described in item 3.2. Subsequently, the immune-related lncRNA–miRNA–mRNA ceRNA network was developed using the R software. The “ggalluvial” package was used to draw a sankey diagram for data visualization.

### Analysis of Immune Cell Infiltration

To estimate the abundance of 22 types of immune cell types in Randall’s plaques and normal–papillary tissue controls, the mRNA microarray dataset GSE73680 was uploaded to the platform of CIBERSORT (http://cibersort.stanford.edu/) (Newman et al., 2015). Only samples that had a CIBERSORT algorithm output of *p* < 0.05 were considered for further analysis. Histograms and heatmaps were drawn to show the rate of immune cell infiltration in different samples. Co-expression patterns in immune-related DE-mRNAs and infiltrating immune cells were analyzed using Pearson’s correlation coefficient. Subsequently, the Wilcoxon rank-sum test was performed to compare differentially infiltrating immune cells between RPs in patients with CaOx stones and controls with normal papillary tissues. The relationship between DE-mRNA expression and the fractions of macrophages and mast cells was also investigated using the Wilcoxon test. Results were visualized using the “heatmap” and “vioplot” packages in the R software.

### Animal Experiments

Thirty male C57BL/6J mice weighing 22–25 g and aging 6–8 weeks were acquired from the Center of Experimental Animals at the Renmin hospital of Wuhan University, Hubei, China. The mice were acclimatized in the animal house of our institution at a steady temperature of 22 ± 2°C and humidity of 40–70% on a 12/12-h light–dark cycle and with free access to water and feed. The animal experiments were conducted according to the Guide for the Care and Use of Laboratory Animals, and the study protocol was approved by the Laboratory Animal Welfare and Ethics Committee of the Renmin hospital of Wuhan University (approval number: WDRM-20200604).

According to previous publications (Okada et al., 2007; Usami et al., 2018), the mice were intraperitoneally injected with 80 or 120 mg/kg of glyoxylate (Sigma–Aldrich; St. Louis, MO, United States) daily for 6 or 12 days to establish a CaOx kidney stone model. Mice were randomly assigned to the five following dosage groups (*n* = 6): control, 80 mg/kg of glyoxylate for 6 days, 120 of mg/kg glyoxylate for 6 days, 80 mg/kg of glyoxylate for 12 days, and 120 mg/kg of glyoxylate for 12 days groups. After 6 or 12 days, the mice were sacrificed, and kidneys were removed for analyses.

### Cell Culture and Treatment

Human renal tubular epithelial cell line (HK-2) cells were provided by Stem Cell Bank, Chinese Academy of Sciences, Shanghai, China. HK-2 cells were cultured in an MEM medium supplemented with 10% fetal bovine serum (Gibco, Waltham, MA, United States) and 1% antibiotics (penicillin/streptomycin). The cells were maintained at 37°C under a humidified atmosphere with 5% CO_2_. Oxalate was purchased from Sigma–Aldrich and dissolved in the culture medium. Subsequently, the cells were cultured in six-well plates, and 0.25 mM or 0.5 mM of oxalate were added for 48 h.

### Hematoxylin and Eosin, Von Kossal, and Masson Staining

After fixation in 4% paraformaldehyde, kidneys were imbedded in paraffin and were cut into 5-μm slices. HE staining was performed to assess the histopathological kidney tubular injuries as previously described (Dong et al., 2019). Injuries were scored as follows: 0, no tubular injury; 1, <10% tubular damage; 2, 10–25% tubular damage; 3, 25–50% tubular damage; 4, 50–74% tubular damage; and 5, >75% tubular damage. Subsequently, crystals were detected using Von Kossal staining, as previously described (Wang et al., 2019). The crystal deposition area was quantified using Image J software. Renal fibrosis was verified using Masson trichrome staining, and the collagen fiber deposition area on kidney sections was quantified using Image J software.

### Immunohistochemistry and Immunofluorescence Staining

The protein expression levels of TGF-β, iNOS, and CD206 were analyzed using immunohistochemical and immunofluorescence staining. Antibodies (i.e., TGF-β [21898-1-AP], iNOS [18985-1-AP], and CD206 [60143-1-Ig]) were purchased from Proteintech (Chicago, IL, United States). All procedures were conducted according to the recommendations of the manufacturer. By comparing the positive area between groups using microscopy, figures were analyzed using Image J software.

### Toluidine Blue Staining

Mast cells were detected using Toluidine blue staining as previously described (Zhang et al., 2017). Mast cells were identified using purple granules, and activated mast cells were characterized by disgorged and loosely packed granules. Activated mast cells per field were counted at a magnification of 400 ×.

### Quantitative Real-Time PCR

Total RNA was extracted from HK-2 cells using TRIzol reagent (Invitrogen Life Technologies, Carlsbad, CA, United States), and RNA purity was measured using spectrophotometry. RNAs were reverse transcribed into cDNAs using the Takara RNA PCR kit (Takara Biotechnology, Shiga, Japan) according to the instructions of the manufacturer. Subsequently, cDNA was amplified by RT-qPCR using an Applied Biosystems SYBR Green mix kit (Applied Biosystems, Foster City, CA, United States). GAPDH was used as an internal reference for lncRNAs and mRNAs, while U6 was used as a reference for miRNA. The primers used for these reactions are shown in Supplementary Table S1. The reactions were measured on the ABI 7900 Real-Time PCR system (Applied Biosystems Life Technologies), and the 2^−ΔΔCT^ method was used for analysis.

### Statistical Analysis

All data are presented as the mean ± SD. Statistical analysis was conducted using SPSS version 19.0 (SPSS Inc., Chicago, IL, United States). Student’s t-test was used to compare differences between groups. A *p*-value of <0.05 was considered statistically significant. All experiments were performed at least three times.

## Results

### Identification of DE mRNAs and lncRNAs

**FIGURE 1:**
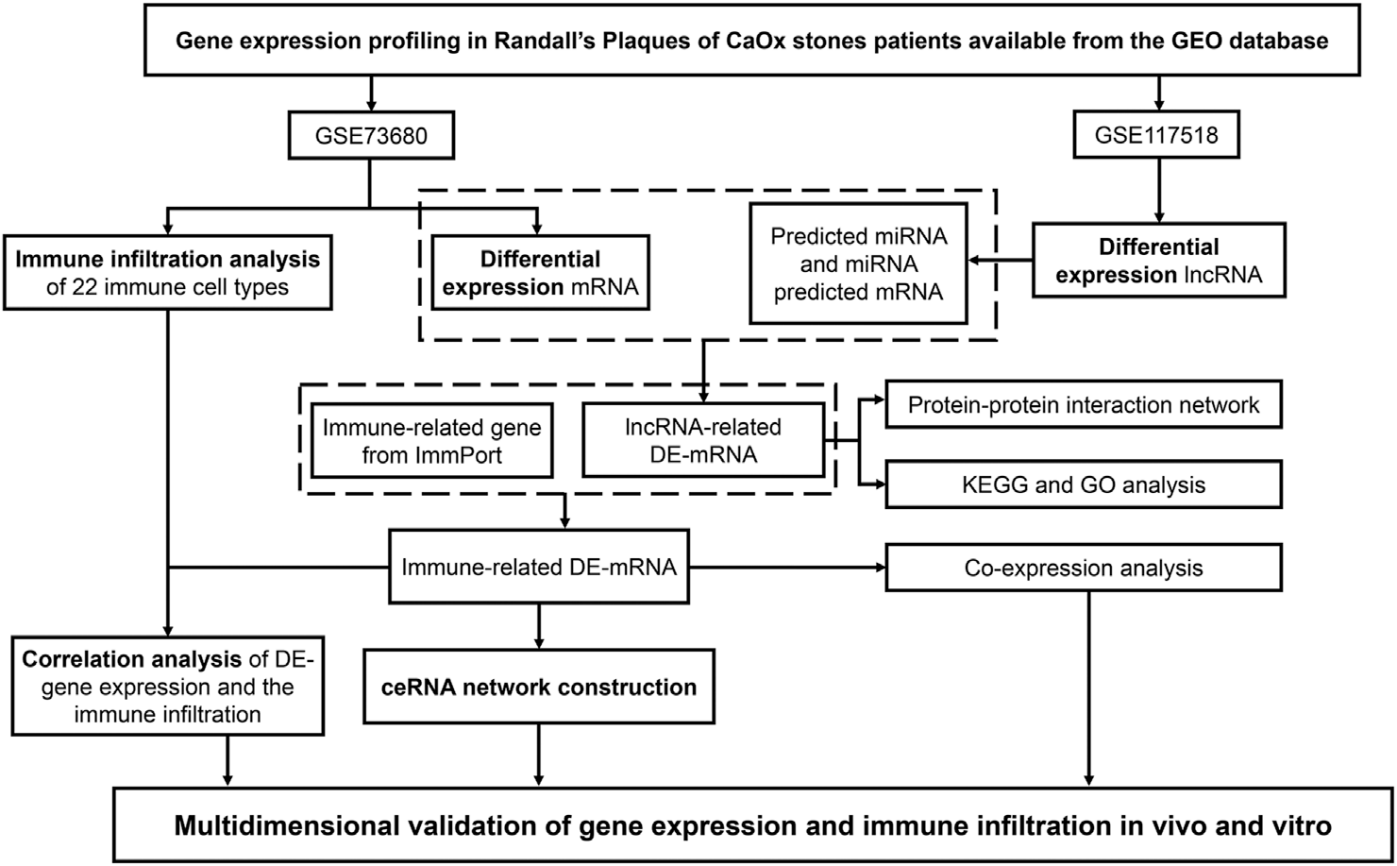
Schematic representation of our analytic process.

**FIGURE 2:**
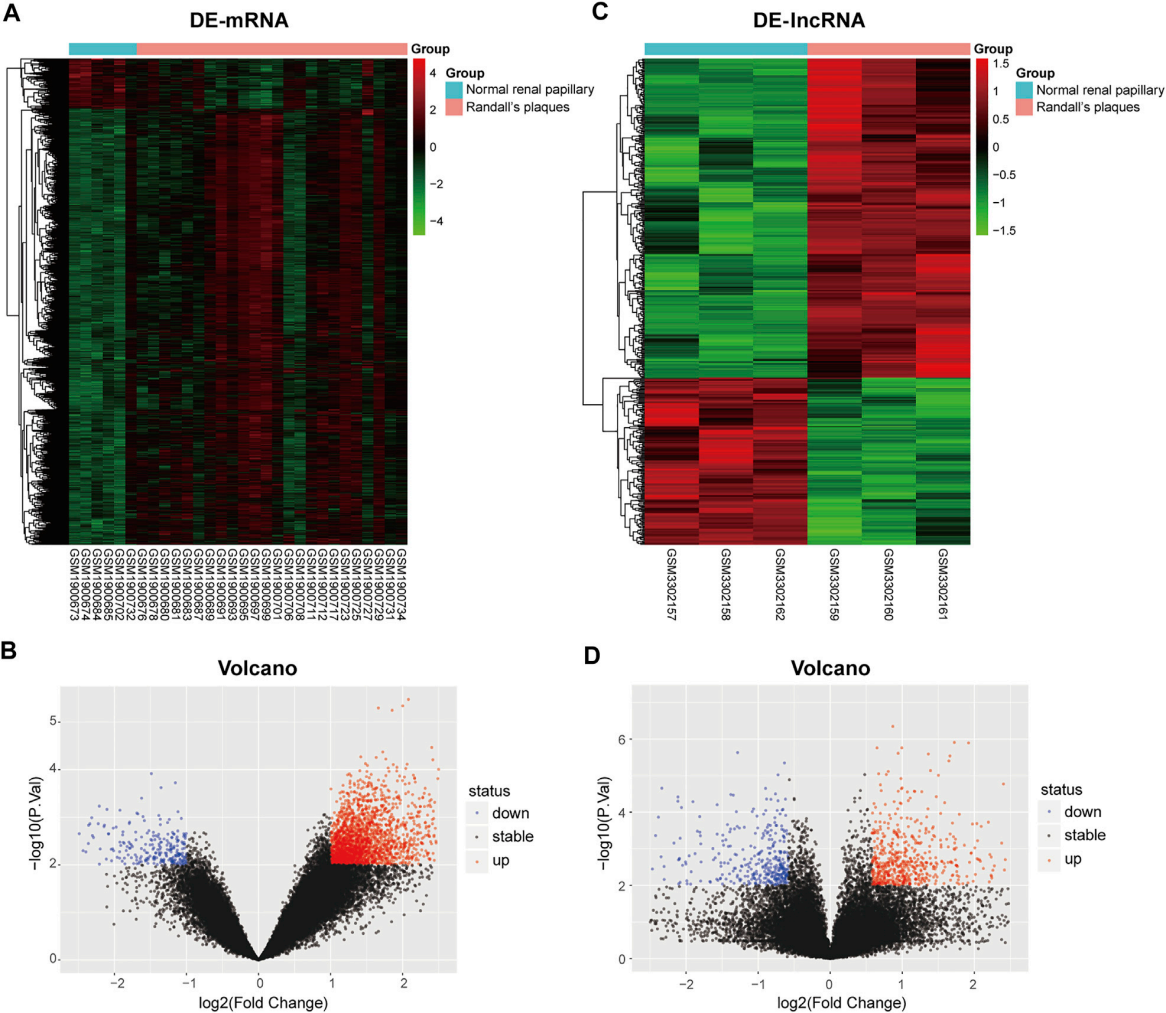
Heatmaps and volcano plots of differentially expressed genes between the Randall’s plaques of patients with CaOx kidney stones and normal–renal papillary tissue controls: **(A,B)** mRNA and **(C,D)** lncRNA.

To clarify the process of this research, a schematic representation is presented in Figure 1. Original data were downloaded from the GSE73680 and GSE117518 datasets in the GEO database. In the GSE73680 dataset, RNA-seq data of 24 RPs from patients with CaOx stones and from six controls with normal papillary tissues were analyzed using criteria of |log_2_FC|>1 and *p* < 0.01. A total of 2,340 DE-mRNAs (2,098 upregulated and 242 downregulated) were compared between RPs and normal papillary tissues. In the GSE117518 dataset, the RNA-seq data of three RPs from patients with CaOx stones and three normal papillary tissues were analyzed using criteria of |log_2_FC|>0.58 and *p* < 0.01. A total of 929 DE-lncRNAs (587 upregulated and 342 downregulated) were identified. Corresponding heatmaps and volcano plots are shown in Figure 2. Details of datasets are presented in Table 1.

### Function Enrichment Analysis of lncRNA-Related DE-mRNAs

**FIGURE 3:**
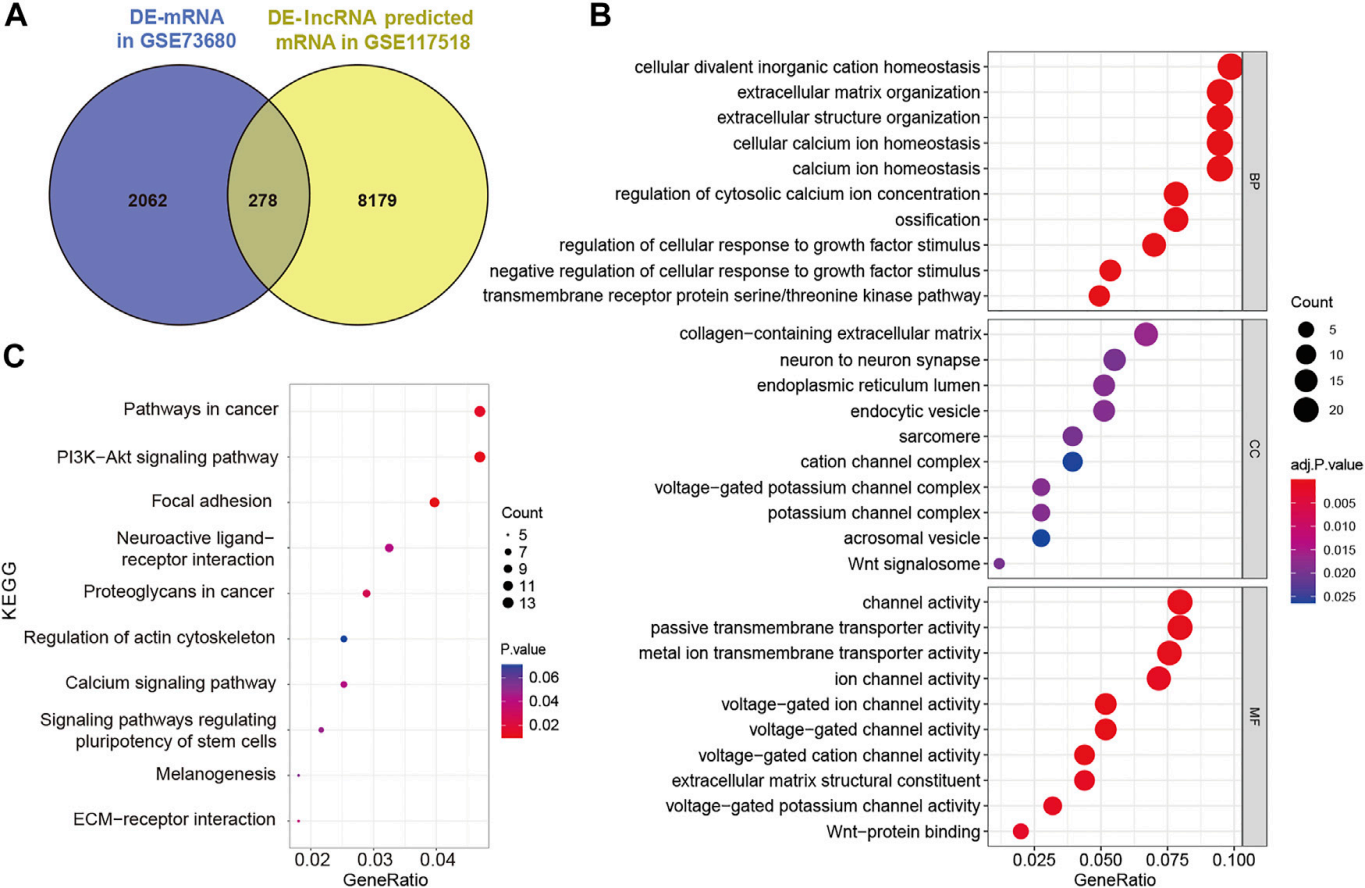
Identification and analysis of lncRNA-related DE-mRNAs. **(A)** Intersection between DE-mRNAs and DE-lncRNAs predicted mRNAs. **(B)** GO enrichment analysis of lncRNA-related DE-mRNAs. **(C)** KEGG enrichment analysis of lncRNA-related DE-mRNAs. DE-mRNAs, differentially expressed mRNAs; DE-lncRNAs, differentially expressed lncRNAs; GO, gene ontology; and KEGG, Kyoto Encyclopedia of Genes and Genomes.

To establish the ceRNA network, DE-lncRNAs were further analyzed. Potential DE-lncRNA-targeted miRNAs were predicted using the miRcode database. Subsequently, miRNA–mRNA pairs were analyzed using the TargetScan, miRTarBase, and miRDB databases. A total of 197 miRNAs and 8,457 mRNAs were predicted. Subsequently, the Venn method was used to analyze the intersection between DE-mRNA and DE-lncRNA-targeted mRNAs (Figure 3A). Consequently, 278 overlapping lncRNA-related DE-mRNAs were identified. To determine the functions of lncRNA-related DE-mRNAs, GO and KEGG enrichment analyses were conducted (Figures 3B,C). A biological process analysis showed that lncRNA-related DE-mRNAs were significantly enriched in extracellular matrix organization, cellular calcium ion homeostasis, and regulation of cellular response to growth factor stimulus. A cellular component analysis showed that lncRNA-related DE-mRNAs were mostly enriched in collagen-containing extracellular matrix and endoplasmic reticulum lumen. A molecular function (MF) analysis showed that lncRNA-related DE-mRNAs were mostly enriched in channel activity and extracellular matrix structural constituent. The KEGG pathway enrichment analysis showed that lncRNA-related DE-mRNAs were significantly enriched in PI3K-Akt signaling pathway, focal adhesion, and extracellular matrix-receptor interaction. Details of GO and KEGG enrichment analyses are presented in Tables 2, 3. The PPI network of lncRNA-related DE-mRNAs is shown in Supplementary Figure S1.

**TABLE 2:** Top 10 GO enrichment terms of differential expression genes.

GO term ID	Term description	GeneRatio	adj.p.val
Biological process			
GO:0072503	Cellular divalent inorganic cation homeostasis	24/243	2.30E-05
GO:0030198	Extracellular matrix organization	23/243	3.25E-06
GO:0043062	Extracellular structure organization	23/243	3.25E-06
GO:0006874	Cellular calcium ion homeostasis	23/243	2.30E-05
GO:0055074	Calcium ion homeostasis	23/243	2.96E-05
GO:0051480	Regulation of cytosolic calcium ion concentration	19/243	9.83E-05
GO:0001503	Ossification	19/243	0.00055
GO:0090287	Regulation of cellular response to growth factor stimulus	17/243	0.00025
GO:0090288	Negative regulation of cellular response to growth factor stimulus	13/243	0.00026
GO:0090101	Negative regulation of transmembrane receptor protein serine/threonine kinase signaling pathway	12/243	8.63E-05
Cellular component			
GO:0062023	Collagen-containing extracellular matrix	17/254	0.01691
GO:0098984	Neuron to neuron synapse	14/254	0.01987
GO:0005788	Endoplasmic reticulum lumen	13/254	0.01863
GO:0030139	Endocytic vesicle	13/254	0.01863
GO:0030017	Sarcomere	10/254	0.01987
GO:0034703	Cation channel complex	10/254	0.02633
GO:0008076	Voltage-gated potassium channel complex	7/254	0.01863
GO:0034705	Potassium channel complex	7/254	0.01863
GO:0001669	Acrosomal vesicle	7/254	0.02647
GO:1990909	Wnt signalosome	3/254	0.01987
Molecular function			
GO:0015267	Channel activity	20/251	0.00085
GO:0022803	Passive transmembrane transporter activity	20/251	0.00085
GO:0046873	Metal ion transmembrane transporter activity	19/251	0.00085
GO:0005216	Ion channel activity	18/251	0.00153
GO:0005244	Voltage-gated ion channel activity	13/251	0.00057
GO:0022832	Voltage-gated channel activity	13/251	0.00057
GO:0022843	Voltage-gated cation channel activity	11/251	0.00057
GO:0005201	Extracellular matrix structural constituent	11/251	0.00148
GO:0005249	Voltage-gated potassium channel activity	8/251	0.00153
GO:0017147	Wnt-protein binding	5/251	0.00236

**TABLE 3:** KEGG pathway enrichment analysis of differentially expressed genes.

KEGG term ID	Term description	Count	p.val
hsa05200	Pathways in cancer	13	0.00692
hsa04151	PI3K-Akt signaling pathway	13	0.00243
hsa04510	Focal adhesion	11	4.70E-04
hsa04080	Neuroactive ligand-receptor interaction	9	0.03502
hsa05205	Proteoglycans in cancer	8	0.01920
hsa04810	Regulation of actin cytoskeleton	7	0.06697
hsa04020	Calcium signaling pathway	7	0.03532
hsa04550	Signaling pathways regulating pluripotency of stem cells	6	0.04222
hsa04916	Melanogenesis	5	0.04751
hsa04512	Extracellular matrix-receptor interaction	5	0.03078

### Construction of the Immune-Related ceRNA Network

**FIGURE 4:**
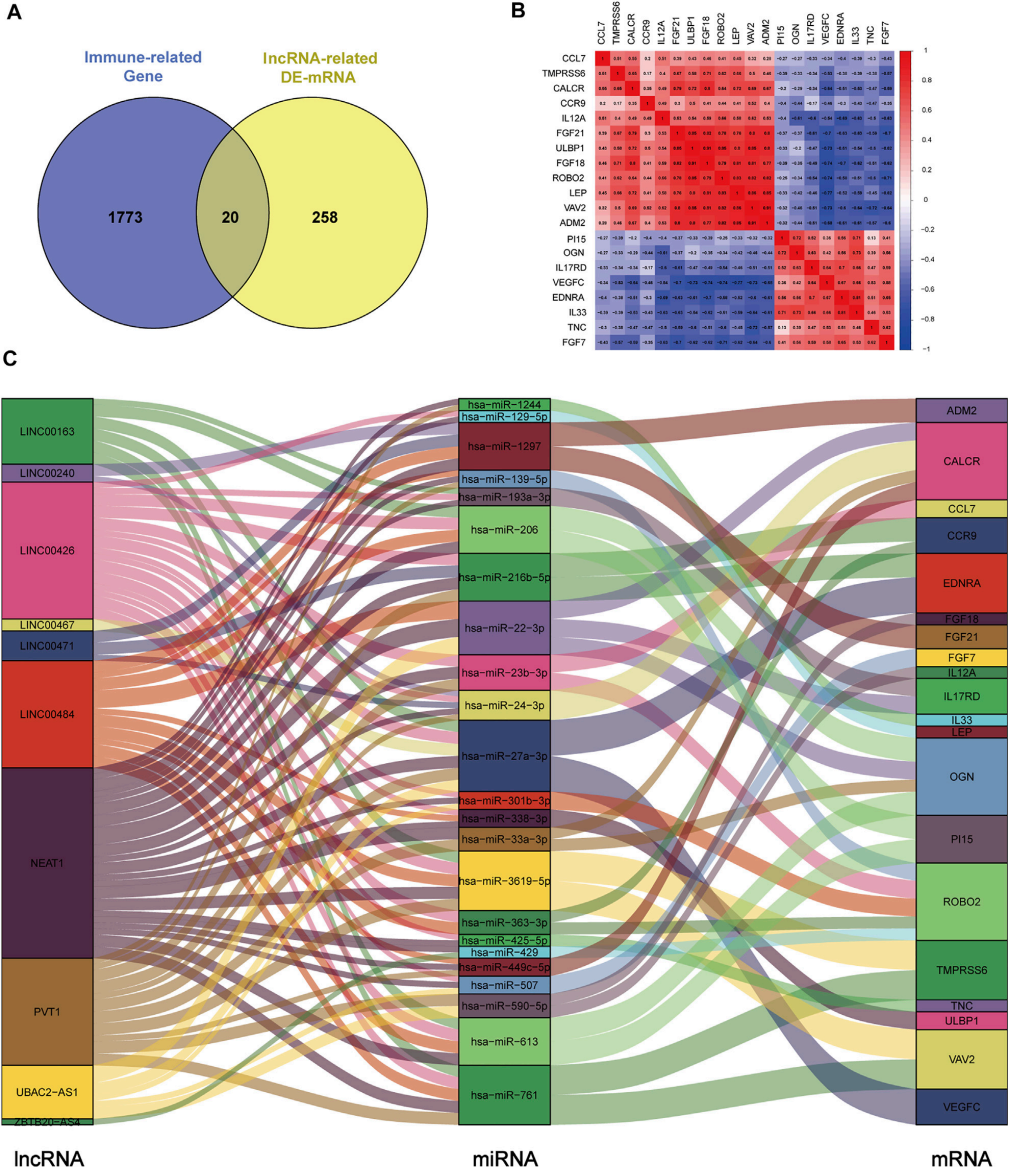
Construction of immune-related ceRNA network in kidney stones. **(A)** Intersection between lncRNA-related DE-mRNAs and immune-related genes. **(B)** Co-expression analysis of immune-related DE-mRNAs. **(C)** Immune-related ceRNA regulatory network.

To construct the immune-related ceRNA network, the Venn method was used to analyze the intersection between lncRNA-related DE-mRNAs and immune-related genes obtained from the IMMPORT database. Consequently, 20 overlapping immune-related DE-mRNAs (12 upregulated and eight downregulated) were identified (Figure 4A). A co-expression analysis of immune-related DE-mRNAs was performed (Figure 4B). Subsequently, immune-related DE-mRNAs and their paired miRNAs and lncRNAs were chosen to develop the ceRNA regulatory network (Figure 4C). In total, the immune-related ceRNA network contained 10 lncRNAs, 23 miRNAs, and 20 mRNAs.

### Composition of Infiltrating Immune Cells

**FIGURE 5:**
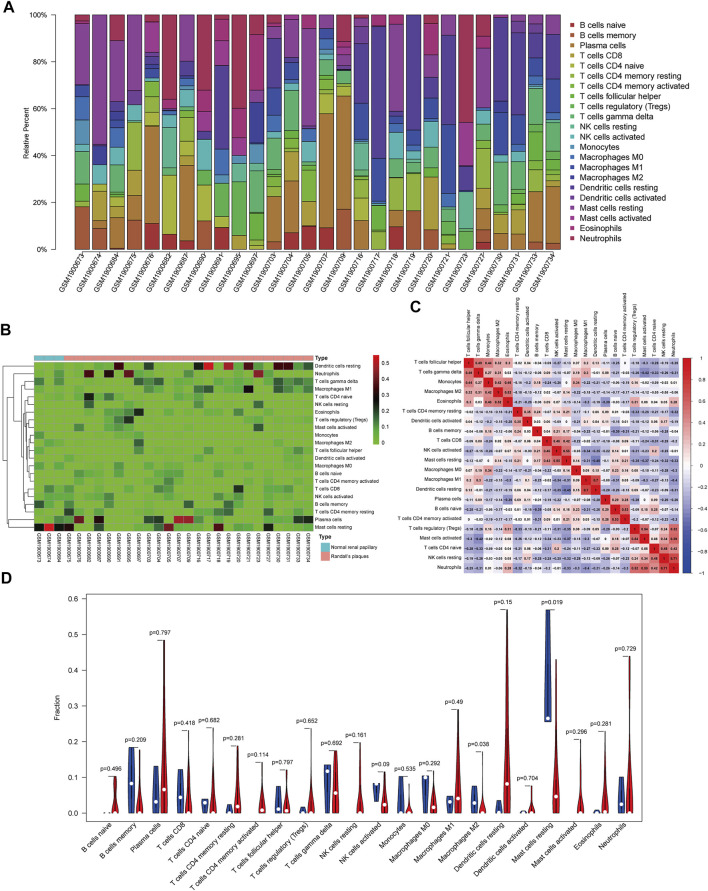
Composition of infiltrating immune cells assessed using the CIBERSORT algorithm in kidney tissues. **(A)** Distribution of immune cell infiltration in each sample. **(B)** Heatmap of immune cell types. **(C)** The correlation among infiltrating immune cells. **(D)** Violin plot of infiltrating immune cells.

The composition of 22 infiltrating immune cells in RPs in patients with CaOx stones and controls with normal papillary tissues were estimated using the CIBERSORT algorithm (Figures 5A,B). The relationships among these 22 immune cells are presented in Figure 5C. M1 macrophages were positively correlated with resting dendritic cells (*R* = 0.70). M2 macrophages were positively correlated with eosinophils (*R* = 0.52). Activated mast cells activated were positively correlated with neutrophils (*R* = 0.59). Resting mast cells were positively correlated with activated NK cells (*R* = 0.55) and negatively correlated with resting dendritic cells (*R* = –0.45). The differential proportion of infiltrating immune cells between RPs in patients with CaOx stones and in controls with normal papillary tissues was analyzed. As shown in Figure 5D, compared with the RPs in controls, M2 macrophages (*p* = 0.038) and resting mast cells (*p* = 0.019) were significantly downregulated and M1 macrophages (*p* = 0.49) and activated mast cells (*p* = 0.296) were significantly upregulated in the RPs in patients with kidney stones.

### Co-Expression Patterns of Infiltrating Immune Cells and DE-mRNAs

**FIGURE 6:**
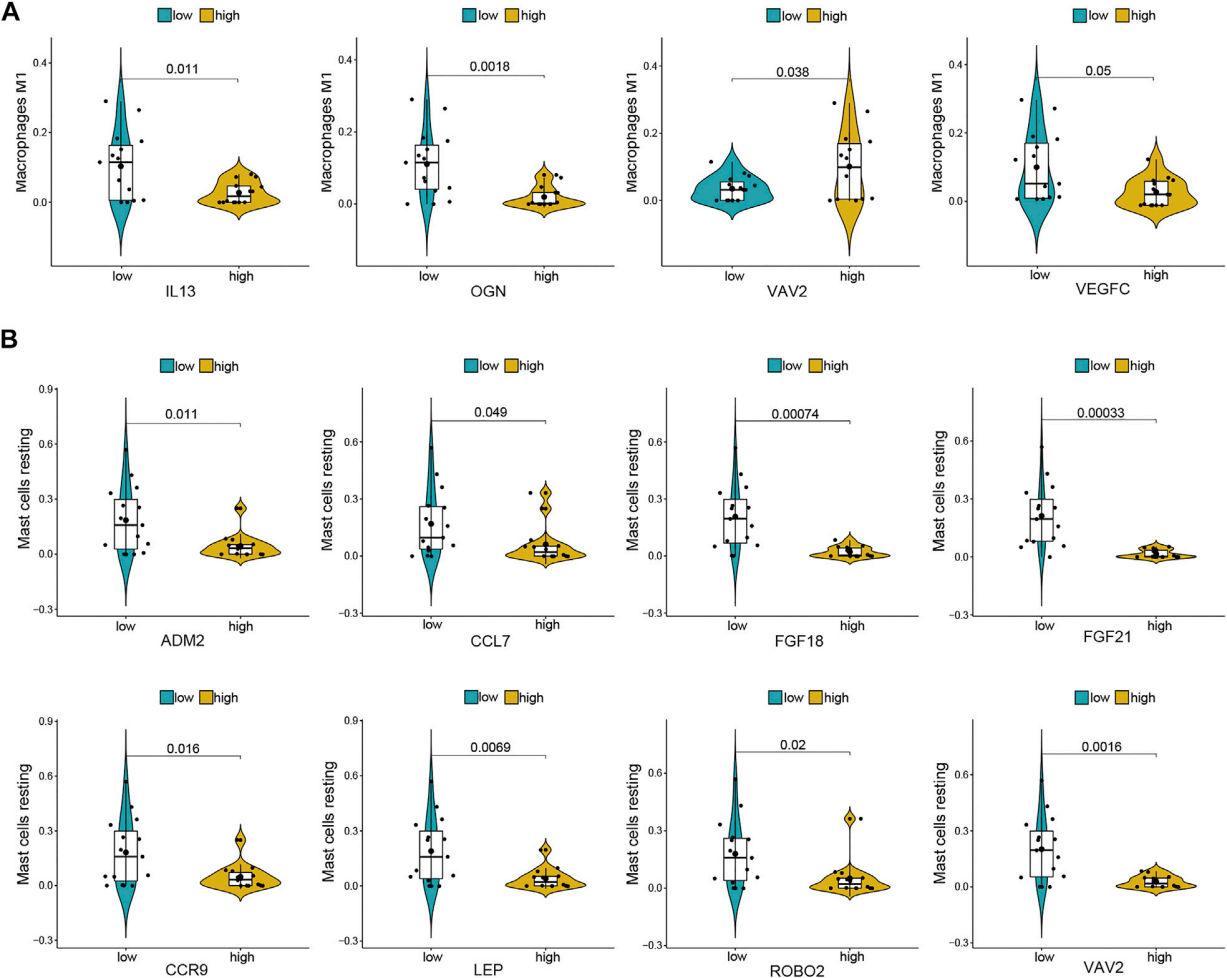
Co-expression patterns of differential infiltrating immune cells and DE-mRNAs. **(A)** Significantly correlated pairs between M1 macrophages and DE-mRNAs. **(B)** Significantly correlated pairs between mast cell resting and DE-mRNAs.

For further analysis, DE-mRNAs were divided to the high expression and low expression groups. The correlation between infiltrating immune cells and DE-mRNAs expression was estimated using the Wilcoxon test, and significantly correlated pairs with *p* < 0.05 are shown in Figure 6. Results indicated that the expression of IL-13, OGN, and VEGFC was significantly negatively correlated with the proportion of M1 macrophages (*p* = 0.011, *p* = 0.002, and *p* = 0.05, respectively), whereas the expression of VAV2 was significantly positively correlated with proportion of M1 macrophages (*p* = 0.038). The expression of ADM2, CCL7, FGF18, FGF21, CCR9, LEP, ROBO2, and VAV2 was significantly negatively correlated with the proportion of resting mast cells (*p* = 0.011, *p* = 0.049, *p* = 0.001, *p* = 0.002, *p* < 0.001, *p* = 0.016, *p* = 0.007, *p* = 0.02, and *p* = 0.002, respectively).

### Validation in a Glyoxylate-Induced Hyperoxaluric Mouse Model

**FIGURE 7:**
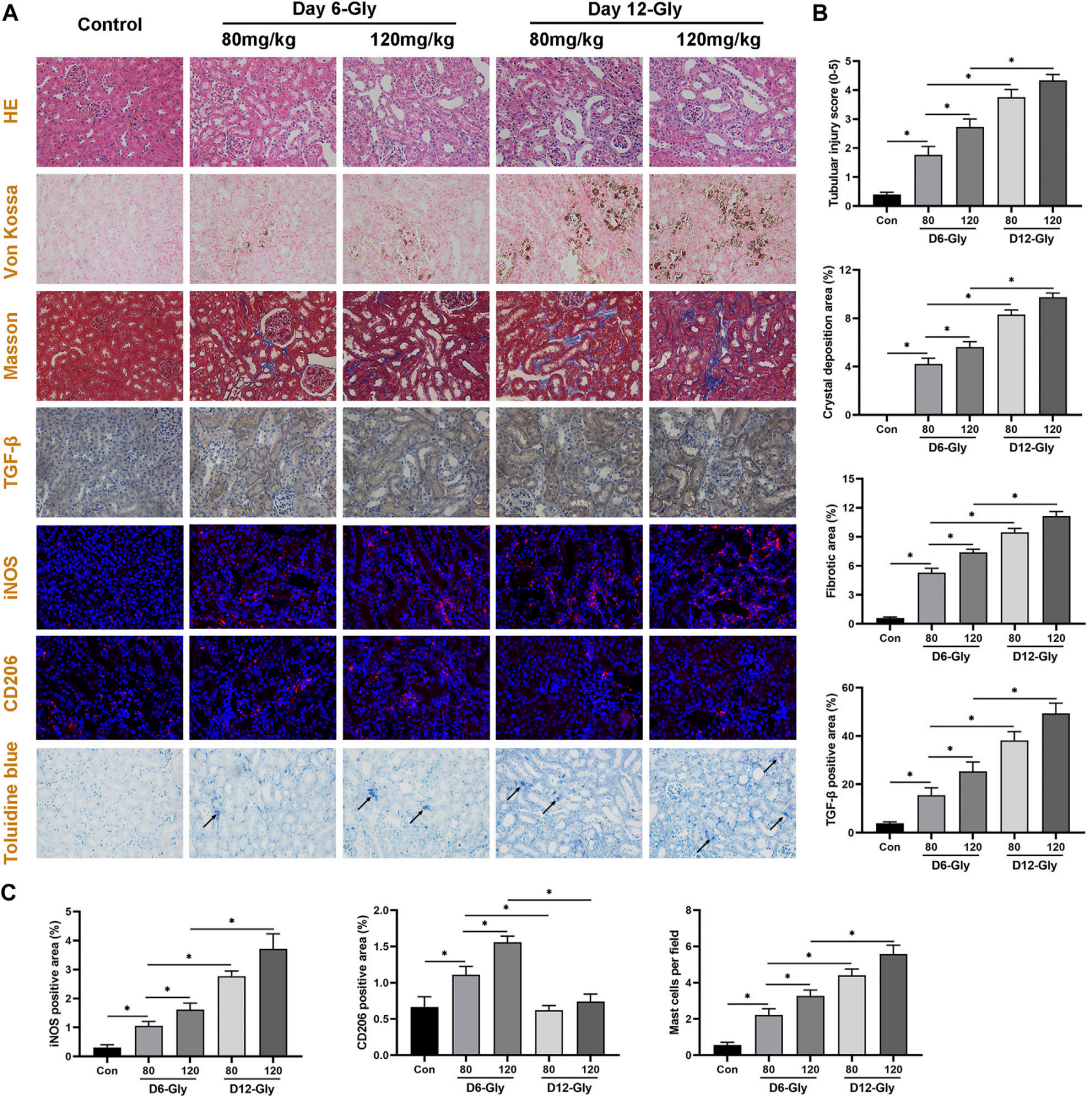
Evaluation of kidney tubular injury, crystal deposition, fibrosis level, macrophage, and mast cell infiltration in a glyoxylate-induced hyperoxaluric mouse model. **(A)** Representative pictures of HE staining, Von Kossal staining, Masson staining, immunohistochemical staining, immunofluorescence staining, and Toluidine blue staining (magnification, 400×). **(B)** Quantification of kidney tubular injury score, CaOx crystal deposition area, collagen fiber deposition area, and TGF-β positive area. **(C)** Quantification of macrophage-related molecules in the iNOS (M1) and CD206 (M2) positive areas, and quantification of activated mast cell per field. Gly, glyoxylate. The arrow indicates activated mast cells. *p < 0.05.

To validate the aforementioned pathway and differentially infiltrating immune cells, kidney tubular injury, crystal deposition, fibrosis level, and macrophage and mast cell infiltration were evaluated in a glyoxylate (Gly)-induced hyperoxaluric mouse model. As shown in Figure 7, kidney tubular injury and crystal deposition were aggravated as treatment concentration and time of glyoxylate increased. Tubular injury and crystals were markedly worse in the day–12 Gly-treated groups than in the day–6 Gly-treated groups. Moreover, tubular injury and crystals were markedly worse in the 120–mg/kg Gly-treated mice than in the 80–mg/kg Gly-treated mice in both day-6 and day-12 groups. Fibrosis and collagen fiber deposition were evaluated using Masson staining and immunohistochemical staining of TGF-β. Results have shown that collagen fiber depositions and TGF-β-positive areas were significantly more frequent in the Gly-treated groups than in the control group in a dose- and time-dependent manner; these results are consistent with those shown in Figure 3B.

Subsequently, the immunofluorescence staining of macrophage-related molecules iNOS (M1) and CD206 (M2) showed that M1 macrophage infiltration significantly increased as kidney stones aggravated, whereas M2 macrophage infiltration increased in the early stages (day 6) and decreased as kidney stones progressed (day 12). Toluidine blue staining showed that activated mast cell infiltration significantly increased in the kidneys of mice with stone formation. As treatment concentration and time of Gly increased, activated mast cells concomitantly increased. These immune cell infiltration results are consistent with our findings shown in Figure 5.

### Construction of Immune-Related hub ceRNA Network and Validation in HK-2 Cells Stimulated With Oxalate

**FIGURE 8:**
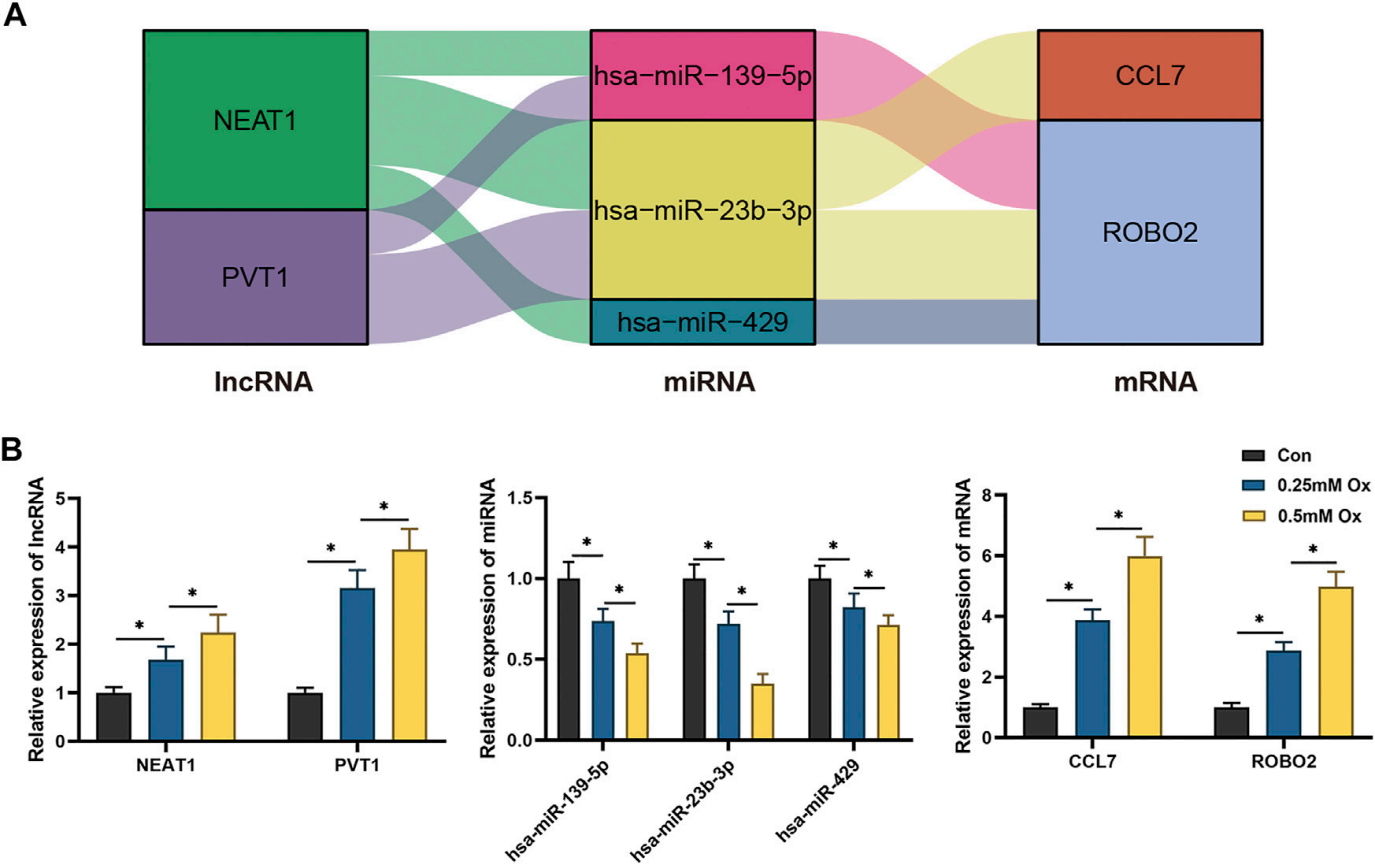
RT-qPCR validation of the immune-related hub ceRNA network in HK-2 cells treated with oxalate. **(A)** The immune-related hub ceRNA network. **(B)** Quantification of the relative expression levels of NEAT1, PVT1, hsa-miR-23b-3p, hsa-miR-429, hsa-miR-139-5p, CCL7, and ROBO2 using RT-qPCR. *p < 0.05.

Through literature review and co-expression analysis of infiltrating immune cells, we established the immune-related hub ceRNA network, comprising 2 lncRNAs, 3 miRNAs, and 2 mRNAs (Figure 8A). Details of the immune-related hub ceRNA network developed from the GSE73680 and GSE117518 datasets are presented in Table 4. To validate the immune-related hub ceRNA network in kidney stones, RT-qPCR was used to detect the expression levels of the hub genes. As shown in Figure 8B, treatment with 0.25 and 0.5 mM oxalate for 48 h significantly upregulated the expression levels of NEAT1, PVT1, CCL7, and ROBO2 but downregulated the expression levels of hsa-miR-23b-3p, hsa-miR-429, and hsa-miR-139-5p in the HK-2 cell line in a dose-dependent manner. These results are consistent with the findings of GEO datasets.

**TABLE 4:** The details of hub ceRNA network from GEO datasets.

LncRNA				microRNA	mRNA			
Name	Fold change	p.val	Status	Name	Name	Fold change	p.val	Status
NEAT1	1.58	0.00053	up	hsa-miR-23b-3p	CCL7	2.96	0.00013	up
					ROBO2	2.27	0.00607	up
				hsa-miR-429	ROBO2	2.27	0.00607	up
PVT1	2.70	0.00043	up	hsa-miR-23b-3p	CCL7	2.96	0.00013	up
					ROBO2	2.27	0.00607	up
				hsa-miR-139-5p	ROBO2	2.27	0.00607	up

## Discussion

Kidney stones are among the most common urological diseases and have a high recurrence rate. In the GEO database, several datasets assessed gene expression profiling by RNA-sequencing in kidney stones. However, most experiments were based on animal models (GSE72135, GSE36446, and GSE75543 datasets) or cell lines (GSE110509, GSE75111, and GSE56934 datasets), rather than patient samples. RPs are considered as the origin of kidney stones (Daudon et al., 2015). Thus, analysis based on the gene expression profiling of RPs may provide more convincing results to reveal kidney stone pathogenesis. In this study, for the first time, an immune-related ceRNA network was constructed and the composition of infiltrating immune cells was estimated based on gene expression profiling in RPs from patients with CaOx kidney stones.

Kidney stones mediate kidney injury and even kidney function loss (Rule et al., 2011). A recent study has found that symptomatic patients with kidney stones have an increased risk off chronic kidney disease compared with the risk of normal individuals (Rule et al., 2009). A retrospective clinical study has demonstrated that 6.01% of patients with kidney stones experience renal atrophy 2 years after percutaneous nephrolithotomy; kidney stones lasting more than 12 months and multiple calyces stone are independent risk factors (Xiangrui et al., 2020), indicating the serious outcomes of kidney stones. The underlying mechanisms may be associated with urinary tract obstruction, infection, and crystal-induced injury and fibrosis (Uribarri, 2020). Convento et al. (2017) demonstrated that the expression levels of TGF-β and epithelial-mesenchymal transition-associated proteins increased in hyperoxaluric mice and HK-2 cells treated with oxalate and CaOx, accompanied by progressive renal failure. In this study, we found that lncRNA-related DE-mRNAs are significantly enriched in extracellular matrix organization, regulation of cellular response to growth factor stimulus, and collagen-containing extracellular matrix, which were associated with kidney interstitial fibrosis. Moreover, we revealed that, throughout kidney stone development, collagen fiber deposition and TGF-β expression were significantly increased in glyoxylate-induced hyperoxaluric mice in a dose- and time-dependent manner. Hence, we speculated that more attention should be paid to kidney stone-induced fibrosis and that lncRNAs may play a crucial role on the corresponding process.

miRNAs, as transcription regulators, are essential in various physiological and pathological processes, including kidney stone-induced renal injury (Jiang et al., 2020; Su et al., 2020). Su et al. (2020) indicated that miR-21 expression increased in hyperoxaluric mice, which promoted CaOx-induced renal tubular injury by PPARA. Jiang demonstrated that miR-155-5p upregulated and promoted oxalate and that CaOx induced oxidative stress injury in HK-2 cells (Jiang et al., 2020). In recent years, the lncRNA–miRNA–mRNA ceRNA network has been proved to be involved in various kidney diseases, including kidney stones (Liang et al., 2019; Liu et al., 2019; Ren et al., 2019). Liu et al. (2019) determined that the interaction between lncRNA H19 and miR-216b facilitated CaOx-induced kidney injury *via* the HMGB1/TLR4/NF-κB pathway. Moreover, Liang et al. (2019) identified the lncRNA–miRNA–mRNA expression variation profile in the urine of patients with CaOx stones. In this study, we constructed an immune-related ceRNA network based on gene expression profiling in RPs, including 10 lncRNAs, 23 miRNAs, and 20 mRNAs, which are potential therapeutic targets. Subsequently, the immune-related hub ceRNA network was established and validated *in vitro*. Treatment with 0.25 and 0.5 mM of oxalate significantly upregulated NEAT1 and PVT1 expression levels and downregulated hsa-miR-23b-3p, hsa-miR-429, and hsa-miR-139-5p expression levels in the HK-2 cell line in a dose-dependent manner. The interaction between NEAT1, PVT1, miR-429, miR-139-5p, and miR-23b-3p may regulate CaOx-induced kidney injury via CCL7 and ROBO2.

The CCL7 gene encodes C-C motif chemokine 7, which can attract monocytes to meditate inflammation and fibrosis (Klein et al., 2009). Inaba et al. (2020) demonstrated that CCL7 increased in a murine model of folic acid-induced acute kidney injury and that the blockade of CCL7 expression reduced monocyte recruitment and ameliorated injury. Sun et al. (2018) reported that CCL7 expression increased in the papillary and urine of patients with nephrolithiasis. The ROBO2 gene encodes the roundabout homolog 2, which is a receptor of slit homolog proteins (SLITs) and is associated with cellular migration guidance (Daehn and Duffield, 2021). ROBO2 dysfunction has been considered to cause congenital kidney and urinary tract abnormalities (Daehn and Duffield, 2021). Moreover, the SLITs/ROBO2 pathway was found to meditate inflammation and acute kidney injury (Chaturvedi and Robinson, 2015). In this study, treatment with 0.25 and 0.5 mM of oxalate significantly upregulated the expression levels of CCL7 and ROBO2 in the HK-2 cell line in a dose-dependent manner, yet the underlying mechanism still needed further investigation.

The polarization of macrophages has been recognized to be involved in the pathogenesis of kidney stones (Taguchi et al., 2021). Taguchi et al. (2016) found that M1-macrophage transfusion promoted kidney stone formation in hyperoxaluric mice and that M2-macrophage transfusion suppressed stone formation. Moreover, Xi et al. (2019) demonstrated that Sirtuin 3-overexpression suppressed crystal deposition through the promotion of the polarization of M2 macrophages. Mast cells have been considered as important components in kidney disease development (Vibhushan et al., 2020). Summers et al. (2012) demonstrated that mast cell activation and degranulation promoted renal fibrosis in mice with unilateral ureteric obstruction, while mast cell-deficient mice showed decreased collagen deposition. Moreover, it has been reported that mast cells can mediate cisplastin-induced acute kidney injury through the recruitment of leukocytes and secretion of TNF (Summers et al., 2011). However, the role of mast cells in the development of kidney stones has not been reported. Consistent with the aforementioned studies, we found that the proportion of M2 macrophages and resting mast cells decreased in the RPs of patients with CaOx stones. Furthermore, throughout kidney stone development, the infiltration of M1 macrophages and activated mast cells increased in mice with glyoxylate-induced hyperoxaluria. M2-macrophage infiltration increased in the early stage and decreased as kidney stones progressed. Together, these results indicate that the polarization of macrophages and recruitment of mast cells may play crucial roles in the development of kidney stones.

This study has several limitations. First, although we analyzed two microarray datasets of kidney stones, the sample size was still limited; this was partly due to the low biopsy rate and low morbidity of kidney stones. Second, no miRNA microarray dataset of patients with kidney stones was available from an open database, thus potential target miRNAs were predicted by online tools. Third, the analysis of infiltrating immune cells only included 22 types; accordingly, the subtypes of macrophages and mast cells require further investigation. Fourth, HK-2 cell line was the only cell line for *in vitro* validation, other renal tubular epithelial cell lines should be studied in the further research. Finally, further functional experiments are needed to demonstrate the mechanisms of the immune-related ceRNA network and their relationship with immune cell infiltration.

In conclusion, in this comprehensive study, we construct an immune-related ceRNA regulatory network and estimate the composition of immune cell infiltration in the RPs of patients with kidney stones. Based on one mRNA and one lncRNA microarray datasets, we identified the DE-mRNA and DE-lncRNA present in RPs and normal papillary tissues and used them for the construction of the ceRNA network. Subsequently, we estimated DE infiltrating immune cells between RPs and normal papillary tissues and their correlation with immune-related DE-mRNAs. Among these cells, macrophages and mast cells were considered to be important immune cells associated with kidney stone formation. Moreover, we validated the ceRNA network and immune infiltration *in vivo* and *in vitro*. These findings provide new insights on the pathogenesis of kidney stones and novel potential therapeutic targets.

## Data Availability

The datasets analyzed for this study can be found in the Gene expression Omnibus (GEO) database (https://www.ncbi.nlm.nih.gov/geo/) (Accession: GSE73680 and GSE117518).

## Supplementary Material

Supplementary Figure 1 PPI network of lncRNA-related differentially expressed mRNAs.

Supplementary Table S1 Sequence of primers used for RT-qPCR.
