# Supplementary material for: Construction and Analysis of Immune Infiltration-Related ceRNA Network for Kidney Stones
Source: Front Genet. 2021 Dec 6;12:774155. doi: 10.3389/fgene.2021.774155 (PMC8686191; doi:10.3389/fgene.2021.774155)
Supplement: Supplementary file 1 [file Table1.docx]

|  |  |  |  |  |  |  |  |  |
| --- | --- | --- | --- | --- | --- | --- | --- | --- |

| Table S1. Sequence of primers used for RT-qPCR. | | |
| --- | --- | --- |
| Gene |  | Sequence (5’ to 3’) |
| LncRNA NEAT1 | Forward | GGGCTCTTCTGGATTTGTTCC |
|  | Reverse | TAGACGCAGCTCAGAACCAA |
| LncRNA PVT1 | Forward | GCTCACAACAGCCTCTGTCT |
|  | Reverse | AAGCTGCCAGATGAGACTCG |
| hsa-miR-23b-3p | Forward | ATTGCCAGGGATTACCACGT |
|  | Reverse | AGTGCAGGGTCCGAGGTATT |
| hsa-miR-429 | Forward | TGTCTGGTAAAACCGTGTCG |
|  | Reverse | AGTGCAGGGTCCGAGGTATT |
| hsa-miR-139-5p | Forward | CACGTGTCTCCAGTGTCGTA |
|  | Reverse | AGTGCAGGGTCCGAGGTATT |
| U6 | Forward | GCTTCGGCAGCACATATACTAAAAT |
|  | Reverse | CGCTTCACGAATTTGCGTGTCAT |
| CCL7 | Forward | TTGCTCAGCCAGTTGGGATT |
|  | Reverse | AAGATTACAGCTTCCCGGGG |
| ROBO2 | Forward | GTTTGTGTTGCGAGGAACTATCT |
|  | Reverse | GTTTTGTCGGAAGTCATCTCGTA |
| GAPDH | Forward | GATTTGGTCGTATTGGGCGC |
|  | Reverse | TTCCCGTTCTCAGCCTTGAC |
|  |  |  |
|  |  |  |
