# Supplementary figures and images for: Construction and Analysis of Immune Infiltration-Related ceRNA Network for Kidney Stones
Source: Front Genet. 2021 Dec 6;12:774155. doi: 10.3389/fgene.2021.774155 (PMC8686191; doi:10.3389/fgene.2021.774155)

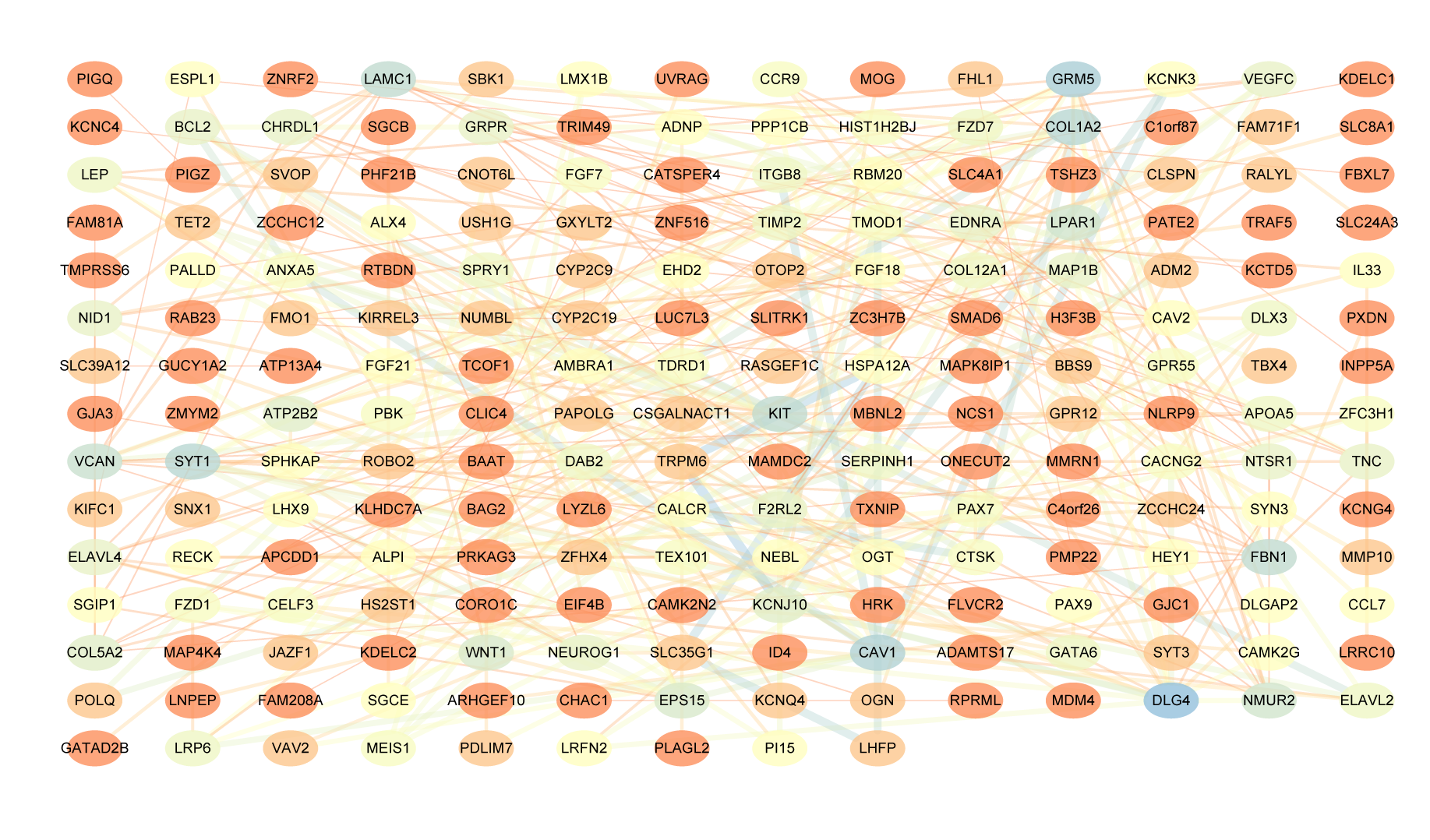

Supplement: Supplementary file 2 [file Image1.TIF]
